# Leaky intestine and impaired microbiome in an amyotrophic lateral sclerosis mouse model

**DOI:** 10.14814/phy2.12356

**Published:** 2015-04-06

**Authors:** Shaoping Wu, Jianxun Yi, Yong-guo Zhang, Jingsong Zhou, Jun Sun

**Affiliations:** 1 Department of Biochemistry, Rush University Medical CenterChicago, Illinois; 2 Department of Physiology, Kansas City University of Medicine and BioscienceKansas, Missouri

**Keywords:** Antimicrobial peptides, autophagy, cell biology, dysbiosis, intestinal permeability, microbiome, Paneth cells, signal transduction, tight junction, ZO-1

## Abstract

Emerging evidence has demonstrated that intestinal homeostasis and the microbiome play essential roles in neurological diseases, such as Parkinson's disease. Amyotrophic lateral sclerosis (ALS) is a fatal neurodegenerative disease characterized by a progressive loss of motor neurons and muscle atrophy. Currently, there is no effective treatment. Most patients die within 3–5 years due to respiratory paralysis. Although the death of motor neurons is a hallmark of ALS, other organs may also contribute to the disease progression. We examined the gut of an ALS mouse model, G93A, which expresses mutant superoxide dismutase (SOD1^G93A^), and discovered a damaged tight junction structure and increased permeability with a significant reduction in the expression levels of tight junction protein ZO-1 and the adherens junction protein E-cadherin. Furthermore, our data demonstrated increased numbers of abnormal Paneth cells in the intestine of G93A mice. Paneth cells are specialized intestinal epithelial cells that can sense microbes and secrete antimicrobial peptides, thus playing key roles in host innate immune responses and shaping the gut microbiome. A decreased level of the antimicrobial peptides defensin 5 alpha was indeed found in the ALS intestine. These changes were associated with a shifted profile of the intestinal microbiome, including reduced levels of *Butyrivibrio Fibrisolvens, Escherichia coli*, and *Fermicus*, in G93A mice. The relative abundance of bacteria was shifted in G93A mice compared to wild-type mice. Principal coordinate analysis indicated a difference in fecal microbial communities between ALS and wild-type mice. Taken together, our study suggests a potential novel role of the intestinal epithelium and microbiome in the progression of ALS.

## Introduction

The intestinal microbiome community modulates numerous aspects of human physiology and is a critical factor in the development of chronic diseases (Turnbaugh et al. 2007; Zhang et al. 2013a; David et al. 2014; Sun and Chang 2014). Intestinal epithelial cells are consistently exposed to bacteria, a process which plays a key role in development, renewal, and immunity (Farhadi et al. 2003; Shen and Turner 2006; Ling et al. 2008; Rajapaksa et al. 2010). Frequent microbial challenges to epithelial cells trigger discrete signaling pathways, promoting molecular changes, such as the secretion of cytokines and chemokines, and alterations of molecules displayed at the epithelial cell surface. Emerging evidence has demonstrated that intestinal homeostasis and the microbiome play essential roles in neurological diseases, such as Parkinson's disease and autism (Forsyth et al. 2011; Hsiao et al. 2013).

Amyotrophic lateral sclerosis (ALS) is a fatal neurodegenerative disease characterized by the progressive loss of motor neurons. Most patients die within 3–5 years due to respiratory paralysis (Alonso et al. 2009). Currently, there is no intervention that can significantly change the course of the disease (Joyce et al. 2011). Thus, there is an urgent need to develop entirely novel interventions to alleviate the disease progression and improve the quality of life for ALS patients. Although the death of motor neurons is a hallmark of ALS, other organs may also contribute to the disease progression. A major event in ALS onset and progression is the motor neuron withdrawal from skeletal muscle. Our previous studies (Zhou et al. 2010; Yi et al. 2011; Luo et al. 2013; Xiao et al. 2015) have reported a defective crosstalk between muscle and motor neurons during ALS progression and demonstrated that the skeletal muscle may actively contribute to disease onset and progression. Here, we ask whether other organs outside of the neuromuscular system may also contribute to ALS progression. The gut is considered “the second brain of the human being” as it contains roughly the same amount of neurons as the spinal cord. The gut–neuron axis is a bidirectional communication system. Intestinal functions are largely controlled by the autonomic and enteric nervous systems (Pfeiffer 2003). The integrative function of gut-secreted hormones, endocrine factors, or bacterial products plays an important role in the physiology and pathophysiology of neurological and psychiatric diseases. We propose that intestinal and microbial homeostasis may play a role in ALS similar to that observed in autism and Parkinson's disease (Forsyth et al. 2011; Hsiao et al. 2013). However, the role of intestinal homeostasis and the gut microbiome in ALS has not been explored.

Many cases of familial ALS (20–25%) are associated with mutations in the superoxide dismutase 1 gene (*SOD1*; Pasinelli and Brown 2006). G93A mice harbor human ALS-causing SOD1 mutations that recapitulate the neuronal and muscle impairment of human ALS patients. These mice have been extensively used to investigate the pathogenesis of ALS and for trials of new therapeutics (Gurney et al. 1994; Joyce et al. 2011). In the current study, we hypothesized that an aberrant microbiome and disrupted intestinal homeostasis are associated with ALS progression. We studied the microbiome and intestinal function of ALS transgenic mice (G93A; Gurney et al. 1994), and found that young G93A mice had damaged intestinal structure, increased intestinal permeability, and abnormal Paneth cells. These changes occur in young G93A mice before disease onset. G93A mice showed an abnormal intestinal microbiome. Our data provide the first evidence of a leaky intestine and impaired microbiome that may play a potential role in ALS disease progression.

## Materials and Methods

### Animals

G93A (Gurney et al. 1994) and the age-matched wild-type mice were used in this study. All experiments were carried out in strict accordance with the recommendation in the Guide for the Care and Use of Laboratory Animals of the National Institutes of Health. The protocol was approved by the IACUC of Rush University.

### Western blot analysis and antibodies

Mouse intestinal mucosal cells were collected by scraping from mouse colon, including proximal and distal colon, as previously described (Liao et al. 2008; Wu et al. 2014). Briefly, mouse mucosal cells were lysed in lysis buffer (1% Triton X-100 (×100; Sigma-Aldrich, St. Louis, MO), 150 mmol/L NaCl (J.T. Baker 3624-19), 10 mmol/L Tris (Fisher Scientific, Waltham, MA, BP152-5) pH 7.4, 1 mmol/L EDTA (Fisher Scientific, BP120-1), 1 mmol/L EGTA (Sigma-Aldrich, E3889) pH 8.0, 0.2 mmol/L sodium ortho-vanadate (Sigma-Aldrich, S6508) and protease inhibitor cocktail (Roche Diagnostics, Nutley, NJ, 118367001). Cultured cells were rinsed twice in ice-cold Hanks’ balanced salt solution (Sigma-Aldrich, H1387), lysed in protein loading buffer (50 mmol/L Tris, pH 6.8, 100 mmol/L dithiothreitol [Amresco, Solon, OH, 0281], 2% SDS [Sigma-Aldrich, L3771], 0.1% bromophenol blue [IBI Scientific, Peosta, IA, IB74040], and 10% glycerol [Sigma-Aldrich, G5516]) and sonicated (Branson Sonifier, Danbury, CT 250). An equal amount of protein was separated by SDS-polyacrylamide gel electrophoresis, transferred to nitrocellulose (Bio-Rad, Hercules, CA, 162-0112), and immunoblotted with primary antibodies: Occludin (Zymed, Las Condes, Santiago, Chile, 33-1500), ZO-1 (Life Technologies, Carlsbad, CA, 33-9100), E-cadherin (BD, Franklin Lakes, NJ, 610405), or *β*-actin (Sigma-Aldrich, A1978) antibodies and visualized by ECL chemiluminescence (Thermo Scientific, Waltham, MA, 32106). Membranes probed with more than one antibody were stripped before reprobing. Western blot bands were quantified using Image Lab 4.01 (Bio-Rad).

### Immunofluorescence

Intestinal tissues were freshly isolated and paraffin-embedded after fixation with 10% neutral-buffered formalin. Immunofluorescence was performed on paraffin-embedded sections (5 *μ*m). After preparation of the slides as described previously (Wu et al. 2014), tissue samples were incubated with anti-lysozyme (Santa Cruz, Dallas, TX, sc27958), or ZO-1 at 4°C overnight. Samples were then incubated with sheep anti-goat Alexa Fluor 594 (Life Technologies, A11058), or goat anti-mouse Alexa Flour 488 (Life Technologies, A-11001), and DAPI (Life Technologies, D1306) for 1 h at room temperature. Tissues were mounted with SlowFade (Life Technologies, s2828), followed by a coverslip, and the edges were sealed to prevent drying. Specimens were examined with Zeiss laser scanning microscope (LSM) 710.

### Fluorescence permeability *in vivo*

Fluorescein Dextran (Molecular weight 3000 Da, diluted in Hanks) was gavaged (50 mg/g mouse). Four hours later, mouse blood samples were collected by cardiac puncture. Fluorescence intensity of the plasma was measured on a fluorescent plate reader, as previously described (Liao et al. 2008; Zhang et al. 2013b).

### Paneth cells counting

Paneth cells in mouse ileal cells were counted after anti-lysozyme immunofluorescence staining. The patterns of lysozyme expression in Paneth cells were classified into two categories: normal and abnormal. Abormal Paneth cells include disordered, depleted, and diffuse ones according to the published methods (Cadwell et al. 2008; Wu et al. 2014).

### Il-17 ELISA

Mouse blood samples were collected by cardiac puncture and placed in tubes containing EDTA (10 mg/mL; Liao et al. 2008). Mouse intestinal mucosal cells were collected by scraping from mouse small intestine and were lysed in lysis buffer, equal amount of protein was used to detect cytokines. Cytokines were measured using a mouse cytokine 20-Plex Panel kit (Life Technologies, LMC0006) according to the manufacturer's instructions. The cytokines included IL-17. Cytokines were analyzed with the Luminex detection system (PerkinElmer CS1000 Autoplex Analyzer).

### Real-time quantitative PCR

Total RNA was extracted from mouse ileal epithelial cells or cultured cells using TRIzol reagent (Life Technologies, 15596-02). RNA was first reverse-transcribed into cDNA with the iScript cDNA synthesis kit (Bio-Rad, 170-8891) according to the manufacturer's manual. The RT-cDNA reaction products were subjected to quantitative real-time PCR using CTFX 96 Real-time system (Bio-Rad, C1000) and SYBR green supermix (Bio-Rad, 172–5124) according to the manufacturer's directions. All expression levels were normalized to *β*-actin levels of the same sample. Percent expression was calculated as the ratio of the normalized value of each sample to that of the corresponding untreated control cells. All real-time PCR reactions were performed in triplicate. Primers were designed as described (Wu et al. 2014).

### Real-time PCR measurement of bacterial DNA

DNA was extracted as previously described (Wu et al. 2014). 16S rDNA PCR reactions were performed with the following primers: Universal bacteria (forward: 5′-TCCTACGGGAGGCAGCAGT-3′; reward: 5′-GGACTACCAGGGTATCTAATCCTGTT-3′), *Escherichia coli* (forward: 5′-CCTACGGGAGGCAGCAGT-3′; reward: 5′-CGTTTAC GGCGTGGACTAC-3′), *B*. *fragilis* (forward: 5′-GGCGCACGGGTG-AGTAACA-3′; reward: 5′-CAATATTCCTC ACTGCTGC-3′), and *Butyrivibrio fibrisolvens* (forward: 5′-CT AACACATGCAAGTCGAACG-3′; reward: 5′-CCGTGTCTCAGTCCCAAT G-3′), The relative amount of 16S rDNA in each sample was estimated using the ΔΔ*C*_*T*_.

### Fecal microbiome 454 pyrosequencing

Fecal microbiome sequencing was done as previously described (Wu et al. 2014). In short, prepared the tubes for microbial sampling with autoclave and then irradiated with ultraviolet light. Collected fecal and placed into the specially prepared tubes. The samples were kept at low temperature with dry ice and mailed to Research and Testing Laboratory, Lubbock, TX, for 454 pyrosequencing. Principal coordinates analysis (PCoA) of unweighted UniFrac distances was plotted using quantitative insights into microbial ecology (QIIME). To determine differences in microbiota composition among the animal groups, the analysis of similarities (ANOSIM) function in the statistical software package PRIMER 6 (PRIMER-E Ltd., Lutton, UK) was used on the unweighted UniFrac distance.

### Statistical analysis

Data are expressed as mean ± SD. Differences between two groups were analyzed by Student's *t* test. *P* values of 0.05 or less were considered statistically significant.

## Results

### Disrupted junction structure in the intestine of G93A mice

**Figure 1:**
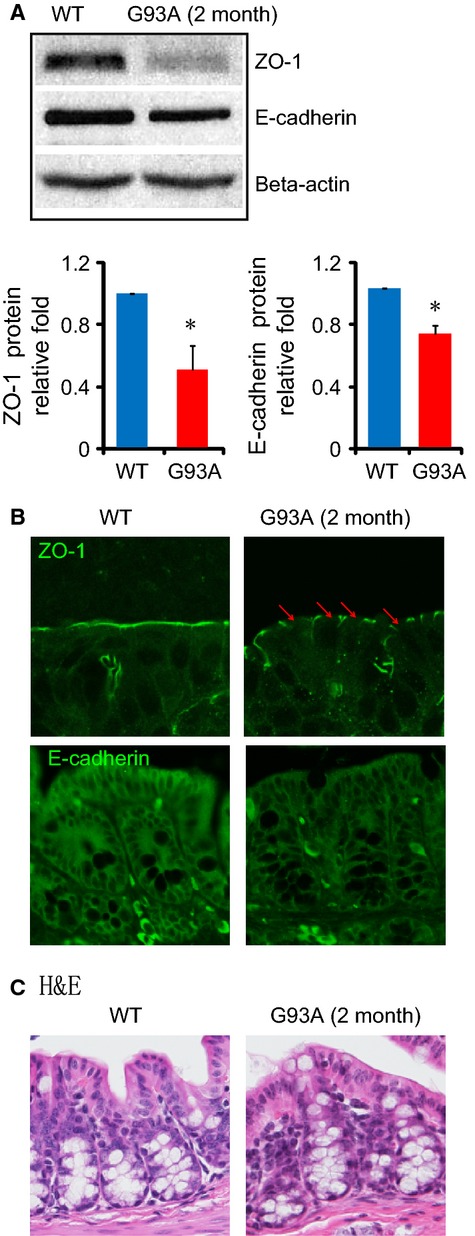
Disrupted intestinal junctions in the 2-month-old amyotrophic lateral sclerosis (ALS) mice. (A) Western blots of ZO-1 and E-cadherin in the colon. The relative band intensities of ZO-1 and E-cadherin are presented as the means ± SD (*n* = 3 per group, *P < 0.05). Western blot bands were quantified using Image Lab 4.01 (Bio-Rad). (B) Immunostaining of ZO-1 and E-cadherin in the colon. The tight junction protein ZO-1 shows discontinuity in the colon of ALS model mice. (C) H&E staining of the colon. Data are from a single experiment and are representative of *n* = 3 mice per group.

G93A mice are asymptomatic until after 3 months of age (Gurney et al. 1994). To test whether the GI abnormality occurs before ALS symptom onset, we used 2-month-old G93A mice. An important component of the intestinal structure is the intercellular junction between the epithelial cells, namely tight junctions (TJs) and adherens junctions (AJs). An AJ is a cell junction in which the cytoplasmic face is linked to the actin cytoskeleton. Internal epithelial cells often rely on TJs and AJs to seal the paracellular space and regulate the permeability of the mucosal barrier. Using Western blot analysis, we found that ZO-1 protein expression was significantly decreased in the G93A gut compared to the WT mice (Fig.1A). The AJ protein E-cadherin in the intestine was also reduced in the G93A mice compared to the WT mice (Fig.1A). Our immunostaining data further showed an abnormal distribution of the TJ protein ZO-1 in the membrane of intestinal epithelial cells in ALS mice at the age of 2 months (Fig.1B ZO1, indicated by arrows). In contrast, ZO-1 was restricted to cellular borders and distributed in a smooth and organized pattern at the apical side of colon the wild-type (WT) mice. Weaker staining of E-cadherin was found in the G93A mice than in the WT mice, which was consistent with the reduced E-cadherin expression at the protein level in the G93A mice. Although there was less E-cadherin in the G93A mice, it was still evenly distributed in colon (Fig.1B). We observed the H&E staining of the intestine of G93A mice at 2 months of age before the onset of disease (Fig.1C). Interestingly, we did not find significant pathological changes in the colon of G93A mice. We studied the expression and distribution of ZO-1 and E-cadherin in colon because colon harbors the most abundant microflora and allows us to study the host–bacteria interactions. We did not find obvious pathological changes in the small intestine of G93A mice by H&E (data not shown). Taken together, these data showed a significant reduction in the ZO-1 and E-cadherin proteins in the ALS model, and those changes could occur before the onset of ALS neuromuscular symptoms.

### G93A mice had increased inflammatory cytokines and intestinal permeability

**Figure 2:**
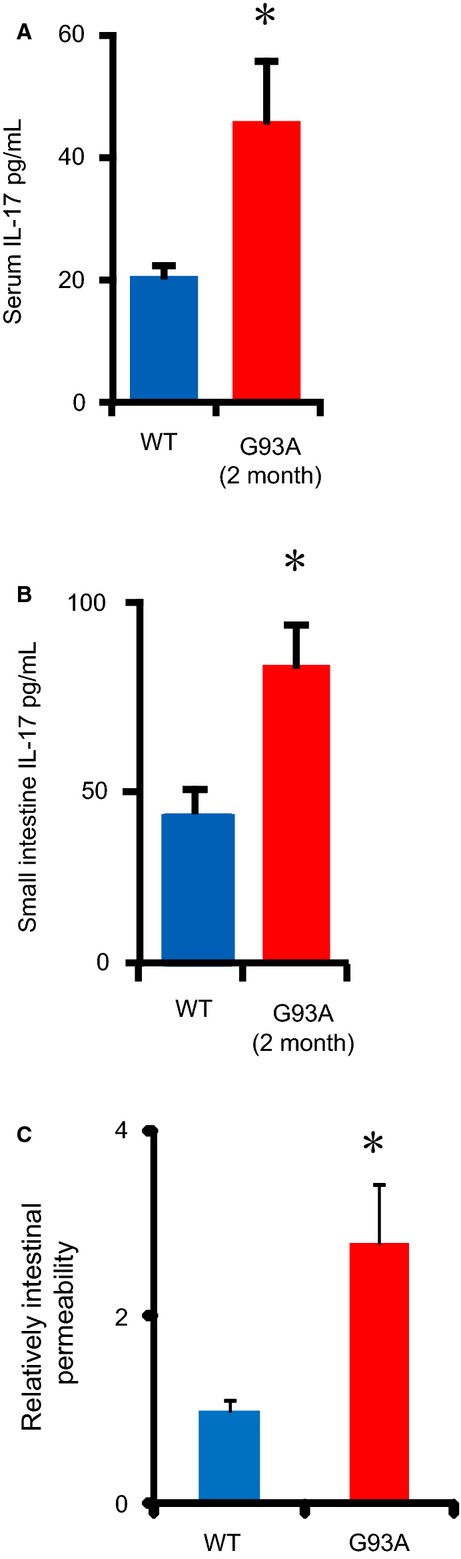
Enhanced IL-17 and intestinal permeability in amyotrophic lateral sclerosis (ALS) mice. (A, B) The inflammatory cytokine IL-17 in mouse serum and the small intestine (*n* = 3, *P < 0.05). (C) Intestinal permeability is increased in ALS mice (*n* = 3, *P < 0.05).

The TJ structure plays a critical role in the intestinal barrier and inflammation (Farhadi et al. 2003; Shen and Turner 2006; Blikslager et al. 2007; Laukoetter et al. 2007; Ling et al. 2008; Rajapaksa et al. 2010). We then collected blood and small intestinal tissues to measure the inflammatory cytokine IL-17 by ELISA. As shown in Figure2A, we observed significantly increased serum IL-17 levels in the young G93A mice (2-month-old). Moreover, IL-17 was enhanced in the intestine in G93A mice (Fig.2B). These data indicate a preinflammatory state in the ALS mice before the onset of disease.

Immunofluorescence-tagged FITC-dextran was also administered by gavage to each mouse for the permeability assay (Fig.2C). Mouse serum was collected to measure the intensity of fluorescence. Higher FITC readings indicated higher permeability of the intestine. We observed a twofold increase in the fluorescence reading in G93A mouse serum compared to WT mouse serum. Overall, the *in vivo* data demonstrate significantly increased inflammatory cytokine IL-17 levels and altered intestinal integrity (leaky gut) in ALS mice.

### Abnormal Paneth cells in G93A mice

**Figure 3:**
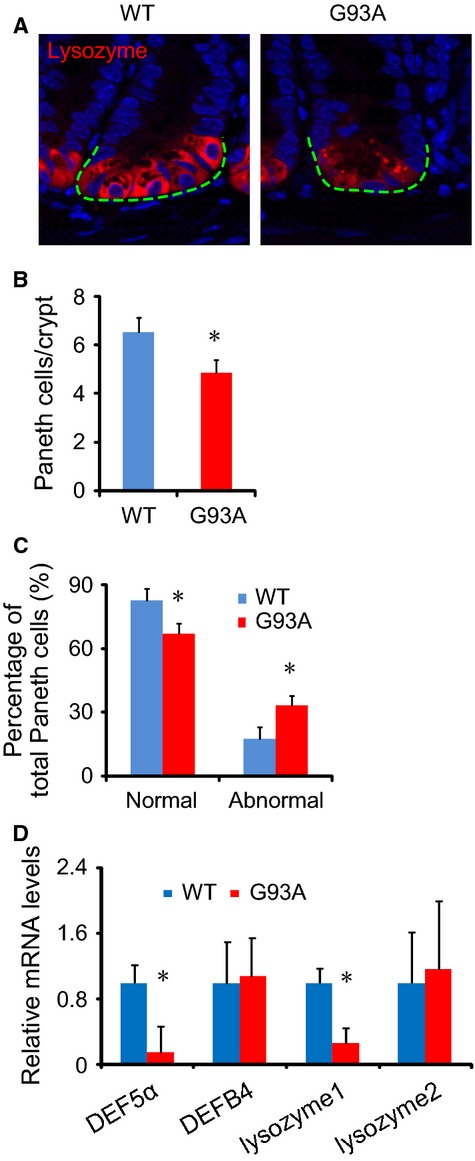
Defects in Paneth cells in the small intestine of amyotrophic lateral sclerosis (ALS) mice. (A) Lysozyme staining in the small intestine of ALS mice. (B) Number of Paneth cells/crypt in the small intestine. Ten slides from each mouse were counted. (C) Percentage of normal and abnormal Paneth cells in ALS mice (*n* = 3/group, *P < 0.05). (D) Defensin 5 alpha mRNA level in 3-month-old mice with symptoms (*n* = 3, *P < 0.05).

Paneth cells are specialized epithelial cells in the small intestine that regulate autophagy activity and host–bacterial interactions in the gut (Schulz et al. 2014; Wu et al. 2014). Lysozymes are components and markers of the Paneth cell secretory granule. Abnormal Paneth cells show disorganized or diminished granules that exhibit diffuse cytoplasmic lysozyme staining (Cadwell et al. 2008). We specifically noticed the decreased number of normal Paneth cells (Fig.3A), shown as the number of Paneth cells per crypt (Fig.3B) in G93A mice. The percentage of abnormal Paneth cells was significantly increased in G93A mice (Fig.3C). The granules of Paneth cells contain antimicrobial peptides (AMPs). A decreased amount of the AMP defensin 5 alpha was also found in the G93A intestine of 3-month-old mice with symptoms (Fig.3D). In contrast, the other AMPs, such as defensin 4 beta, were not changed in the G93A intestine. Paneth cells are associated with autophagic activity in the intestine. Furthermore, we found a significant reduction in lysozyme 1 in the G93A intestine, although lysozyme two was not changed. These data indicate a potential dysfunction of Paneth cells and autophagy in the intestine of G93A mice.

### Abnormal intestinal microbiome in G93A mice

**Figure 4:**
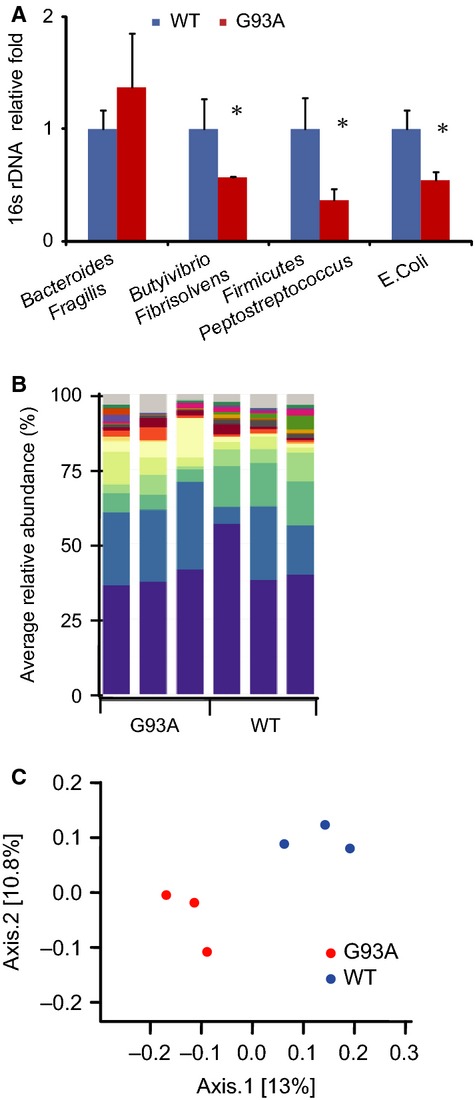
Shift of fecal microbial communities in amyotrophic lateral sclerosis (ALS) mice. (A) Butyrivibrio Fibrisolvens was decreased in G93A amyotrophic lateral sclerosis model mice. Real-time PCR of the bacterial universal 16S rRNA gene and 16S rRNA for *Escherichia coli, Bacteroides Fragilis*, and *Butyrivibrio Fibrisolvens* in fecal samples from both G93A and wild-type mice. Primers specific to universal 16S rRNA were used as an endogenous control to normalize loading between samples. The relative amount of 16S rRNA in each sample was estimated using the ΔΔ*C*_*T*_ method (*n* = 3, *P < 0.05). (B) *Bacterial* community of fecal samples from ALS and WT mice using 454 16S rRNA sequencing data (*n* = 3/group). (C) Principal coordinates analysis (PCoA) of unweighted UniFrac distances of 16S rRNA genes. An analysis was conducted for ALS (red) and WT (blue) mice. The results indicate that fecal microbial communities differ in ALS and WT mice.

The Paneth cells produce IL-17 (Takahashi et al. 2008), sense microbes and secrete AMPs. Although Paneth cells are located in the ileum, AMPs are released to the entire intestine. Enteric AMPs are essential regulators of intestinal microbial ecology (Salzman et al. 2009). Furthermore, we collected fecal samples from G93A mice and tested the bacterial profile by 16rRNA analysis. Our PCR data showed an abnormal intestinal microbiome, in which butyrate-producing bacteria (*Butyrivibrio Fibrisolvens), E. coli,* and *Fermicus*, were reduced (Fig.4A). This change occurs in young ALS mice at the age of 2 months, which is before disease onset. We further collected fecal samples from G93A mice and tested the bacterial profile by 454 16rRNA sequencing. The relative abundance of bacteria was shifted in G93A mice compared to WT mice (Fig.4B). Principal coordinate analysis (PCoA) indicated that fecal microbial communities differ in G93A mice compared to WT mice (Fig.4C). Overall, our data showed a shift in the intestinal microbiome profile in G93A mice.

## Discussion

In the current study, we have demonstrated, for the first time, that the gut of an ALS mouse model (G93A) loses its integrity is unable to maintain its structure and function, and the microbiome profile at the early stage of disease progression is altered. We discovered a damaged TJ structure with increased gut permeability and higher levels of the inflammatory cytokine IL-17. The expression levels of the junction proteins ZO-1 and E-cadherin were significantly reduced in the intestine of G93A mice, which may explain the increased permeability and leakage in the intestine. Furthermore, we found a significantly increased number of abnormal Paneth cells in G93A mice. In accordance with the key roles of Paneth cells in regulating innate immune responses and shaping the gut microbiome, a reduced protein level of the AMP defensin 5 alpha and a shifted profile of the intestinal microbiome were also demonstrated in the intestine of G93A mice. The structural and physiological changes mirror the population shift in the intestinal microbiome (dysbiosis) of G93A mice.

The early changes of the intestinal microbiome and the leaky gut likely promote a systematic inflammatory response. Indeed, inflammation in ALS has been reported in human (McCombe and Henderson 2011). Our data regarding enhanced IL-17 in the blood are in line with the report. Moreover, the increased IL-17 level in the G93A intestine provides new evidence of a link between intestinal inflammation and ALS.

Autophagy is a key process that responds to injury and pathogens in host defense systems (Yuk et al. 2012; Murrow and Debnath 2013) and eliminates misfolded proteins (Klionsky et al. 2008). Dysregulation of autophagy is implicated in ALS (Li et al. 2008; Sasaki 2011; Crippa et al. 2013). A reduced autophagy flux has been reported in G93A motor neurons (Zhang et al. 2014). Our recent study demonstrated a suppressed autophagic response in the skeletal muscle of young G93A mice before disease onset (Xiao et al. 2015). In the intestine, we specifically noticed a decrease in normal Paneth cells and a reduction in lysozyme 1, one of the proteases in lysosomes associated with autophagy maturation (Phan et al. 2009; Virgin and Levine 2009). Decreased levels of the antimicrobial peptide defensin were also found in the G93A intestine. Our data indicate a potential dysfunction of autophagy in the intestine of G93A mice, which may lead to a reduced capacity to eliminate misfolded proteins and promote gut dysfunction in G93A mice.

Protein aggregation of SOD1-mutant protein is a hallmark of ALS pathology (Deng et al. 2006; Ivanova et al. 2014; Kim et al. 2014). The ALS-causing mutation SOD1^G93A^ forms protein aggregates in motor neurons (Deng et al. 2006). Our previous study shows that SOD1^G93A^ forms protein aggregates in skeletal muscle fibers, which lead to mitochondrial dysfunction (Luo et al. 2013). SOD1 gene mutations may also form protein aggregates in the intestine of G93A mice. It has been reported that mice overexpressing the antioxidant enzyme SOD1 have significantly reduced intestinal tissue damage (Lee et al. 2014). Thus, we speculate that SOD1^G93A^ mutations may play an essential role in pathophysiological functions in the intestine. It is possible that G93A mice may develop an age- or stress-dependent phenotype within the GI system. This will be examined in our future study by the evaluation of G93A mice at different stages of development. Understanding the pathogenic mechanism will help to identify new targets for improving therapeutic strategies for ALS.

The gut microbiome plays essential roles in neurological diseases, such as autism and Parkinson's disease (Collins and Bercik 2009; Finegold et al. 2010; Hsiao et al. 2013). A leaky gut with an increased translocation of LPS from gram-negative enterobacteria also plays a role in the inflammatory pathophysiology of depression (Maes et al. 2008). Our data provide evidence regarding dysbiosis, a shift of bacterial populations, in an ALS mouse model. Gut microbial interactions are complex, fluid, and capable of adjusting to physiological perturbations that are encountered on a daily basis. However, selective shifts in the gut microbiota as a consequence of host pathobiology can upset critical intermicrobe as well as host–microbe relationships and initiate pathophysiological processes leading to disease. Two examples of this include the loss of beneficial microbes and their products and the emergence of disease-promoting microbes that produce microbial metabolites and proinflammatory mediators, which negatively impact the intestine and other organ systems (Sun and Chang 2014). We observed a reduction in butyrate-producing bacteria (*Butyrivibrio Fibrisolvens*). These changes occur in young G93A mice before ALS onset. A leaky gut could contribute to the altered microbiome environment that leads to reduced beneficial bacterial products, such as short-chain fatty acids (SCFAs), or enhanced toxic bacterial products, such as LPS. Butyrate is an SCFA that modulates the physiology of the host through binding of G protein-coupled receptors (GPCR; Fung et al. 2012; Miletta et al. 2014). However, to our knowledge, there is no report of bacterial products and their receptors that play a role in neuromuscular degeneration in ALS progression or functional changes in other organs. It is still unknown whether translocation of bacteria and their products/structures can be linked to specific organ dysfunction in ALS. There is no study of ALS genes, such as SOD1, that might be related to intestinal microbiome homeostasis. Our current study indicates that impaired gut-neuromuscular crosstalk may actively contribute to ALS progression. For future studies, we will further define the changes in the intestinal microbiome and function, levels of bacterial products, and receptors of bacterial products during ALS progression and their correlation with the progressive decline of neuromuscular function. Meanwhile, we will define the mechanism of abnormal epithelial junction structure and dysbiosis in ALS mice.

In summary, our current study has identified changes in the gut microbial profile and the level of the inflammatory cytokine IL-17 as well as an abnormal intestinal junction structure and function (permeability and Paneth cells) in an ALS mouse model. Our studies have provided the first insight into the potential contribution of aberrant intestinal homeostasis in ALS progression. ALS patients often come to the clinic only after their disease has become symptomatic, making it difficult to understand the early events leading to the disease. The G93A mouse model allows us to examine the roles of genetic factors and other organs in the early events of disease development and to elucidate the potential pathogenic mechanisms. Our findings may bring novel concepts to the ALS research field for developing potential preventive and therapeutic strategies for combating this devastating disease.

## Conflict of Interest

The authors have no conflict of interest.
